# Supplementary material for: A genetic screen reveals a key role for Reg1 in 2-deoxyglucose sensing and yeast AMPK inhibition
Source: PLoS Genet. 2025 Oct 9;21(10):e1011896. doi: 10.1371/journal.pgen.1011896 (PMC12520357; doi:10.1371/journal.pgen.1011896)
Supplement: S1 Table — (DOCX) [file pgen.1011896.s004.docx]

**Supplementary table S1.** Results of the complementation group analysis of the 2DG-resistant mutants obtained in the screen. Quantification of growth of diploids obtained from the indicated crosses (WT, *hxk2∆,* *glc7-*Q48P and *reg1∆*) on 0.2% 2DG. Colony growth was quantified on ImageJ, relative growth within each row is represented as a heat-map by the shading of the cell (white: growth, black: no growth). CG: assigned complementation group based on quantification as determined in Material and Methods. The “Gene” and “Mutation” columns refer either to the results of the mutation identified by PCR of the indicated gene from gDNA and sequencing, or from whole genome resequencing followed by confirmation by PCR on gDNA and sequencing.

| **Clone** | **CG** | **x WT**  **(BY4742)** | **x *hxk2*∆** | **x *glc7 (Q48P)*** | **x *reg1*∆** | **Gene** | **Mutation** | **Note** |
| --- | --- | --- | --- | --- | --- | --- | --- | --- |
| **Control strains** |  |  |  |  |  |  |  |  |
| WT |  |  |  |  |  |  |  | Haploinsufficiency of *REG1* confers partial 2DG resistance [1] |
| *hxk2*∆ |  |  |  |  |  | *HXK2* | ∆ |  |
| *reg1*∆ |  |  |  |  |  | *REG1* | ∆ |  |
| *glc7*-E241K |  |  |  |  |  | *GLC7* | E241K |  |
| **Mutants** |  |  |  |  |  |  |  |  |
| 1.4 | *REG1* |  |  |  |  | *REG1* | D147N |  |
| 1.5 | *REG1* |  |  |  |  | *REG1* | R513* |  |
| 1.9 | *REG1* |  |  |  |  | *REG1* | Y403* | Identified by whole-genome resequencing and confirmed. |
| 1.16 | *REG1* |  |  |  |  | *REG1* | W165G |  |
| 1.17 | *REG1* |  |  |  |  | *REG1* | K281E |  |
| 1.18 | *REG1* |  |  |  |  | *REG1* | V233E |  |
| 1.22 | *REG1* |  |  |  |  | *REG1* | W151* |  |
| 2.1 | *REG1* |  |  |  |  | *REG1* | V349* |  |
| 2.3 | *REG1* |  |  |  |  | *REG1* | V688* |  |
| 2.7 | *REG1* |  |  |  |  |  |  |  |
| 2.16 | *REG1* |  |  |  |  | *REG1* | K175T |  |
| 2.20 | *REG1* |  |  |  |  | *REG1* | S547* |  |
| 3.7 | *REG1* |  |  |  |  | *REG1* | V349* |  |
| 3.11 | *REG1* |  |  |  |  | *REG1* | I279M |  |
| 3.14 | *REG1* |  |  |  |  | *REG1* | D661N |  |
| 3.15 | *REG1* |  |  |  |  | *REG1* | A54T |  |
| 3.16 | *REG1* |  |  |  |  | *REG1* | T67N |  |
| 4.1 | *REG1* |  |  |  |  | *REG1* | T450* |  |
| 4.4 | *REG1* |  |  |  |  | *REG1* | H694fs |  |
| 4.7 | *REG1* |  |  |  |  | *REG1* | R72G |  |
| 4.11 | *REG1* |  |  |  |  | *REG1* | R72G |  |
| 4.14 | *REG1* |  |  |  |  | *REG1* | W165* |  |
| 4.15 | *REG1* |  |  |  |  | *REG1* | S66* |  |
| 4.19 | *REG1* |  |  |  |  |  |  |  |
| 4.20 | *REG1* |  |  |  |  | *REG1* | L294* |  |
| 4.22 | *REG1* |  |  |  |  | *REG1* | W165* |  |
| 5.1 | *REG1* |  |  |  |  | *REG1* | Q655* |  |
| 5.4 | *REG1* |  |  |  |  | *REG1* | L228I |  |
| 5.5 | *REG1* |  |  |  |  | *REG1* | P797S |  |
| 5.7 | *REG1* |  |  |  |  | *REG1* | M443* |  |
| 5.9 | *REG1* |  |  |  |  | *pREG1* | A-166G | Mutation in the *REG1* promoter (-166bp before ATG) |
| 5.11 | *REG1* |  |  |  |  |  |  |  |
| 5.18 | *REG1* |  |  |  |  | *GLC7* | E125* |  |
| 6.1 | *REG1* |  |  |  |  | *REG1* | V349* |  |
| 6.2 | *REG1* |  |  |  |  | *REG1* | E473* |  |
| 6.3 | *REG1* |  |  |  |  | *REG1* | M1* |  |
| 6.4 | *REG1* |  |  |  |  | *REG1* | E473* |  |
| 6.5 | *REG1* |  |  |  |  | *REG1* | Y640* | Identified by whole genome resequencing and confirmed. |
| 6.6 | *REG1* |  |  |  |  | *REG1* | K65* |  |
| 6.7 | *REG1* |  |  |  |  | *REG1* | Q293* |  |
| 6.9 | *REG1* |  |  |  |  |  |  |  |
| 6.16 | *REG1* |  |  |  |  |  |  |  |
| 6.17 | *REG1* |  |  |  |  | *REG1* | K175T |  |
| 6.20 | *REG1* |  |  |  |  | *REG1* | N624S | Identified by whole genome resequencing and confirmed. |
| 7.2 | *REG1* |  |  |  |  | *REG1* | M1R |  |
| 7.3 | *REG1* |  |  |  |  | *REG1* | N21fs |  |
| 7.6 | *REG1* |  |  |  |  | *REG1* | G609* |  |
| 7.1 | *REG1* |  |  |  |  | *REG1* | S546* |  |
| 7.10 | *REG1* |  |  |  |  | *REG1* | L228I |  |
| 7.11 | *REG1* |  |  |  |  | *REG1* | E298* |  |
| 7.13 | *REG1* |  |  |  |  | *REG1* | Y640* |  |
| 7.14 | *REG1* |  |  |  |  | *REG1* | P278Q |  |
| 7.15 | *REG1* |  |  |  |  | *REG1* | P278R |  |
| 7.18 | *REG1* |  |  |  |  | *pREG1* | G-113A | Mutation in the *REG1* promoter (-113bp before ATG) |
| 9.1 | *REG1* |  |  |  |  | *REG1* | Q398* |  |
| 9.7 | *REG1* |  |  |  |  | *GAL83* | N239K | Identified by whole genome resequencing and confirmed. |
| 9.9 | *REG1* |  |  |  |  | *REG1* | E195D |  |
| 9.11 | *REG1* |  |  |  |  | *REG1* | E195Q |  |
| 9.13 | *REG1* |  |  |  |  | *REG1* | I279T |  |
| 9.19 | *REG1* |  |  |  |  | *REG1* | L194F |  |
| 10.4 | *REG1* |  |  |  |  | *REG1* | Y640* |  |
| 10.5 | *REG1* |  |  |  |  | *REG1* | F468C |  |
| 10.6 | *REG1* |  |  |  |  | *REG1* | Y640* |  |
| 10.8 | *REG1* |  |  |  |  |  |  |  |
| 10.10 | *REG1* |  |  |  |  | *DCK1* | T592S | Identified by whole genome resequencing and confirmed. |
| 10.12 | *REG1* |  |  |  |  | *REG1* | N503ins | 20 bp tandem duplication (ATGTTGATGTTAATAACAAT) |
| 10.13 | *REG1* |  |  |  |  | *REG1* | Y561* |  |
| 10.14 | *REG1* |  |  |  |  | *REG1* | M1I |  |
| 10.17 | *REG1* |  |  |  |  | *REG1* | S725* |  |
| 10.20 | *REG1* |  |  |  |  | *DCK1* | T592S | Identified by whole genome resequencing and confirmed. |
| 11.1 | *REG1* |  |  |  |  | *REG1* | Q398* |  |
| 11.3 | *REG1* |  |  |  |  | *REG1* | W227* |  |
| 1.1 | *HXK2* |  |  |  |  |  |  |  |
| 1.2 | *HXK2* |  |  |  |  |  |  |  |
| 1.3 | *HXK2* |  |  |  |  | *HXK2* | Y221* |  |
| 1.7 | *HXK2* |  |  |  |  |  |  |  |
| 1.10 | *HXK2* |  |  |  |  |  |  |  |
| 1.12 | *HXK2* |  |  |  |  |  |  |  |
| 1.14 | *HXK2* |  |  |  |  |  |  |  |
| 1.15 | *HXK2* |  |  |  |  | *HXK2* | L310F |  |
| 1.19 | *HXK2* |  |  |  |  |  |  |  |
| 1.21 | *HXK2* |  |  |  |  |  |  |  |
| 2.5 | *HXK2* |  |  |  |  |  |  |  |
| 2.9 | *HXK2* |  |  |  |  |  |  |  |
| 2.11 | *HXK2* |  |  |  |  |  |  |  |
| 2.13 | *HXK2* |  |  |  |  |  |  |  |
| 2.14 | *HXK2* |  |  |  |  |  |  |  |
| 2.17 | *HXK2* |  |  |  |  |  |  |  |
| 2.19 | *HXK2* |  |  |  |  |  |  |  |
| 2.21 | *HXK2* |  |  |  |  |  |  |  |
| 3.1 | *HXK2* |  |  |  |  |  |  |  |
| 3.2 | *HXK2* |  |  |  |  |  |  |  |
| 3.3 | *HXK2* |  |  |  |  |  |  |  |
| 3.4 | *HXK2* |  |  |  |  |  |  |  |
| 3.5 | *HXK2* |  |  |  |  |  |  |  |
| 3.9 | *HXK2* |  |  |  |  |  |  |  |
| 3.12 | *HXK2* |  |  |  |  |  |  |  |
| 3.13 | *HXK2* |  |  |  |  |  |  |  |
| 3.18 | *HXK2* |  |  |  |  |  |  |  |
| 3.19 | *HXK2* |  |  |  |  |  |  |  |
| 3.20 | *HXK2* |  |  |  |  |  |  |  |
| 4.3 | *HXK2* |  |  |  |  |  |  |  |
| 4.5 | *HXK2* |  |  |  |  | *HXK2* | Y241D |  |
| 4.9 | *HXK2* |  |  |  |  |  |  |  |
| 4.16 | *HXK2* |  |  |  |  |  |  |  |
| 4.18 | *HXK2* |  |  |  |  |  |  |  |
| 4.21 | *HXK2* |  |  |  |  |  |  |  |
| 5.3 | *HXK2* |  |  |  |  |  |  |  |
| 5.6 | *HXK2* |  |  |  |  |  |  |  |
| 5.10 | *HXK2* |  |  |  |  |  |  |  |
| 5.12 | *HXK2* |  |  |  |  |  |  |  |
| 5.13 | *HXK2* |  |  |  |  |  |  |  |
| 5.14 | *HXK2* |  |  |  |  |  |  |  |
| 5.19 | *HXK2* |  |  |  |  |  |  |  |
| 6.10 | *HXK2* |  |  |  |  |  |  |  |
| 6.12 | *HXK2* |  |  |  |  |  |  |  |
| 6.15 | *HXK2* |  |  |  |  |  |  |  |
| 6.18 | *HXK2* |  |  |  |  |  |  |  |
| 7.4 | *HXK2* |  |  |  |  |  |  |  |
| 7.8 | *HXK2* |  |  |  |  |  |  |  |
| 7.9 | *HXK2* |  |  |  |  |  |  |  |
| 7.12 | *HXK2* |  |  |  |  |  |  |  |
| 7.17 | *HXK2* |  |  |  |  | *HXK2* | Q251* |  |
| 7.20 | *HXK2* |  |  |  |  |  |  |  |
| 7.21 | *HXK2* |  |  |  |  |  |  |  |
| 9.2 | *HXK2* |  |  |  |  |  |  |  |
| 9.3 | *HXK2* |  |  |  |  |  |  |  |
| 9.4 | *HXK2* |  |  |  |  |  |  |  |
| 9.5 | *HXK2* |  |  |  |  |  |  |  |
| 9.6 | *HXK2* |  |  |  |  |  |  |  |
| 9.15 | *HXK2* |  |  |  |  |  |  |  |
| 9.16 | *HXK2* |  |  |  |  |  |  |  |
| 10.1 | *HXK2* |  |  |  |  |  |  |  |
| 10.2 | *HXK2* |  |  |  |  |  |  |  |
| 10.3 | *HXK2* |  |  |  |  |  |  |  |
| 10.7 | *HXK2* |  |  |  |  |  |  |  |
| 10.9 | *HXK2* |  |  |  |  |  |  |  |
| 10.11 | *HXK2* |  |  |  |  |  |  |  |
| 10.15 | *HXK2* |  |  |  |  |  |  |  |
| 10.16 | *HXK2* |  |  |  |  |  |  |  |
| 10.18 | *HXK2* |  |  |  |  | *HXK2* | G307C |  |
| 10.21 | *HXK2* |  |  |  |  |  |  |  |
| 11.4 | *HXK2* |  |  |  |  |  |  |  |
| 1.13 | *GLC7* |  |  |  |  | *GLC7* | N85D |  |
| 1.20 | *GLC7* |  |  |  |  | *GLC7* | N85D |  |
| 5.2 | *GLC7* |  |  |  |  | *GLC7* | Q293P |  |
| 9.10 | *GLC7* |  |  |  |  |  |  |  |
| 2.4 | DOM |  |  |  |  | *SNF4* | P128S | Identified by whole genome resequencing and confirmed. |
| 3.8 | DOM |  |  |  |  | *SNF4* | Y32D | Identified by whole genome resequencing and confirmed. |
| 3.17 | DOM |  |  |  |  | *GAL83* | D225Y |  |
| 4.13 | DOM |  |  |  |  | *SNF4* | S163R |  |
| 5.8 | DOM |  |  |  |  | *SNF4* | L78R |  |
| 5.15 | DOM |  |  |  |  |  |  |  |
| 5.17 | DOM |  |  |  |  | *SNF4* | L78R |  |
| 9.12 | DOM |  |  |  |  | *SNF1* | Y167D |  |
| 9.18 | DOM |  |  |  |  |  |  |  |
| 11.2 | DOM |  |  |  |  |  |  |  |
| 1.6 | N/A |  |  |  |  | *REG1* | W219* |  |
| 1.8 | N/A |  |  |  |  |  |  |  |
| 1.11 | N/A |  |  |  |  |  |  |  |
| 2.2 | N/A |  |  |  |  |  |  |  |
| 2.6 | N/A |  |  |  |  |  |  |  |
| 2.8 | N/A |  |  |  |  |  |  |  |
| 2.10 | N/A |  |  |  |  |  |  |  |
| 2.12 | N/A |  |  |  |  |  |  |  |
| 2.15 | N/A |  |  |  |  |  |  |  |
| 2.18 | N/A |  |  |  |  |  |  |  |
| 2.22 | N/A |  |  |  |  | *ROD1* | L554* | Identified by whole genome resequencing and confirmed. |
| 3.6 | N/A |  |  |  |  |  |  |  |
| 3.10 | N/A |  |  |  |  |  |  |  |
| 3.21 | N/A |  |  |  |  |  |  |  |
| 4.2 | N/A |  |  |  |  |  |  |  |
| 4.6 | N/A |  |  |  |  |  |  |  |
| 4.8 | N/A |  |  |  |  |  |  |  |
| 4.10 | N/A |  |  |  |  |  |  |  |
| 4.12 | N/A |  |  |  |  |  |  |  |
| 4.17 | N/A |  |  |  |  | *CYC8* | W240* |  |
| 5.16 | N/A |  |  |  |  |  |  |  |
| 5.20 | N/A |  |  |  |  |  |  |  |
| 5.21 | N/A |  |  |  |  | *TPS2* | R619C | Identified by whole genome resequencing and confirmed. |
| 6.8 | N/A |  |  |  |  |  |  |  |
| 6.11 | N/A |  |  |  |  |  |  |  |
| 6.13 | N/A |  |  |  |  |  |  |  |
| 6.14 | N/A |  |  |  |  |  |  |  |
| 6.19 | N/A |  |  |  |  |  |  |  |
| 7.7 | N/A |  |  |  |  |  |  |  |
| 7.5 | N/A |  |  |  |  |  |  |  |
| 7.16 | N/A |  |  |  |  | *FYV10* | S388L | Identified by whole genome resequencing and confirmed. |
| 7.19 | N/A |  |  |  |  | *FYV10* | S388L | Identified by whole genome resequencing and confirmed. |
| 9.8 | N/A |  |  |  |  | *GLC7* | Y254C |  |
| 9.14 | N/A |  |  |  |  |  |  |  |
| 9.17 | N/A |  |  |  |  |  |  |  |
| 9.20 | N/A |  |  |  |  |  |  |  |
| 9.21 | N/A |  |  |  |  | *GLC7* | Y254C |  |
| 10.19 | N/A |  |  |  |  |  |  |  |
