# Supplementary material for: A genetic screen reveals a key role for Reg1 in 2-deoxyglucose sensing and yeast AMPK inhibition
Source: PLoS Genet. 2025 Oct 9;21(10):e1011896. doi: 10.1371/journal.pgen.1011896 (PMC12520357; doi:10.1371/journal.pgen.1011896)
Supplement: S2 Table — (DOCX) [file pgen.1011896.s005.docx]

**Supplementary Table S2.** Quantification of growth on various drugs of the 2DG-resistant mutants obtained in the screen. CG: complementation group, as determined in Supplementary table 1; CTRL: control condition (untreated), 2DG: medium containing 0.2% 2DG; SEL: medium containing 200mM sodium selenite; TUN: medium containing 0.5μg/mL Tunicamycin. Colony growth was quantified on ImageJ, relative growth within each row is represented as a heat-map by the shading of the cell (white: growth, black: no growth).

| **Clone** | **CG** | **CTRL** | **2DG** | **SEL** | **TUN** | **Confirmed**  **gene** | **Mutation** | **WGS?** |
| --- | --- | --- | --- | --- | --- | --- | --- | --- |
| WT |  |  |  |  |  |  |  |  |
| *hxk2*∆ |  |  |  |  |  |  |  |  |
| *reg1*∆ |  |  |  |  |  |  |  |  |
| *glc7-Q48P* |  |  |  |  |  |  |  |  |
| 1.1 | *HXK2* |  |  |  |  |  |  |  |
| 1.2 | *HXK2* |  |  |  |  |  |  |  |
| 1.3 | *HXK2* |  |  |  |  | *HXK2* | Y221* |  |
| 1.4 | *REG1* |  |  |  |  | *REG1* | D147N |  |
| 1.5 | *REG1* |  |  |  |  | *REG1* | R513* |  |
| 1.6 |  |  |  |  |  | *REG1* | W219* |  |
| 1.7 | *HXK2* |  |  |  |  |  |  |  |
| 1.8 |  |  |  |  |  |  |  |  |
| 1.9 | *REG1* |  |  |  |  | *REG1* | Y403* | Y |
| 1.10 | *HXK2* |  |  |  |  |  |  |  |
| 1.11 |  |  |  |  |  |  |  |  |
| 1.12 | *HXK2* |  |  |  |  |  |  |  |
| 1.13 | *GLC7* |  |  |  |  | *GLC7* | N85D |  |
| 1.14 | *HXK2* |  |  |  |  |  |  |  |
| 1.15 | *HXK2* |  |  |  |  | *HXK2* | L310F |  |
| 1.16 | *REG1* |  |  |  |  | *REG1* | W165G |  |
| 1.17 | *REG1* |  |  |  |  | *REG1* | K281E |  |
| 1.18 | *REG1* |  |  |  |  | *REG1* | V233E |  |
| 1.19 | *HXK2* |  |  |  |  |  |  |  |
| 1.20 | *GLC7* |  |  |  |  | *GLC7* | N85D |  |
| 1.21 | *HXK2* |  |  |  |  |  |  |  |
| 1.22 | *REG1* |  |  |  |  | *REG1* | W151* |  |
| 2.1 | *REG1* |  |  |  |  | *REG1* | V349* |  |
| 2.2 |  |  |  |  |  |  |  |  |
| 2.3 | *REG1* |  |  |  |  | *REG1* | V688* |  |
| 2.4 | *DOM* |  |  |  |  | *SNF4* | P128S | Y |
| 2.5 | *HXK2* |  |  |  |  |  |  |  |
| 2.6 |  |  |  |  |  |  |  |  |
| 2.7 | *REG1* |  |  |  |  |  |  |  |
| 2.8 |  |  |  |  |  |  |  |  |
| 2.9 | *HXK2* |  |  |  |  |  |  |  |
| 2.10 |  |  |  |  |  |  |  |  |
| 2.11 | *HXK2* |  |  |  |  |  |  |  |
| 2.12 |  |  |  |  |  |  |  |  |
| 2.13 | *HXK2* |  |  |  |  |  |  |  |
| 2.14 | *HXK2* |  |  |  |  |  |  |  |
| 2.15 |  |  |  |  |  |  |  |  |
| 2.16 | *REG1* |  |  |  |  | *REG1* | K175T |  |
| 2.17 | *HXK2* |  |  |  |  |  |  |  |
| 2.18 |  |  |  |  |  |  |  |  |
| 2.19 | *HXK2* |  |  |  |  |  |  |  |
| 2.20 | *REG1* |  |  |  |  | *REG1* | S547* |  |
| 2.21 | *HXK2* |  |  |  |  |  |  |  |
| 2.22 |  |  |  |  |  | *ROD1* | L554* |  |
| 3.1 | *HXK2* |  |  |  |  |  |  |  |
| 3.2 | *HXK2* |  |  |  |  |  |  |  |
| 3.3 | *HXK2* |  |  |  |  |  |  |  |
| 3.4 | *HXK2* |  |  |  |  |  |  |  |
| 3.5 | *HXK2* |  |  |  |  |  |  |  |
| 3.6 |  |  |  |  |  |  |  |  |
| 3.7 | *REG1* |  |  |  |  | *REG1* | V349* |  |
| 3.8 | DOM |  |  |  |  | *SNF4* | Y32D | Y |
| 3.9 | *HXK2* |  |  |  |  |  |  |  |
| 3.10 |  |  |  |  |  |  |  |  |
| 3.11 | *REG1* |  |  |  |  | *REG1* | I279M |  |
| 3.12 | *HXK2* |  |  |  |  |  |  |  |
| 3.13 | *HXK2* |  |  |  |  |  |  |  |
| 3.14 | *REG1* |  |  |  |  | *REG1* | D661N |  |
| 3.15 | *REG1* |  |  |  |  | *REG1* | A54T |  |
| 3.16 | *REG1* |  |  |  |  | *REG1* | T67N |  |
| 3.17 | DOM |  |  |  |  | *GAL83* | D225Y |  |
| 3.18 | *HXK2* |  |  |  |  |  |  |  |
| 3.19 | *HXK2* |  |  |  |  |  |  |  |
| 3.20 | *HXK2* |  |  |  |  |  |  |  |
| 3.21 |  |  |  |  |  |  |  |  |
| 4.1 | *REG1* |  |  |  |  | *REG1* | T450* |  |
| 4.2 |  |  |  |  |  |  |  |  |
| 4.3 | *HXK2* |  |  |  |  |  |  |  |
| 4.4 | *REG1* |  |  |  |  | *REG1* | H694fs |  |
| 4.5 | *HXK2* |  |  |  |  | *HXK2* | Y241D |  |
| 4.6 |  |  |  |  |  |  |  |  |
| 4.7 | *REG1* |  |  |  |  | *REG1* | R72G |  |
| 4.8 |  |  |  |  |  |  |  |  |
| 4.9 | *HXK2* |  |  |  |  |  |  |  |
| 4.10 |  |  |  |  |  |  |  |  |
| 4.11 | *REG1* |  |  |  |  | *REG1* | R72G |  |
| 4.12 |  |  |  |  |  |  |  |  |
| 4.13 | DOM |  |  |  |  | *SNF4* | S163R |  |
| 4.14 | *REG1* |  |  |  |  | *REG1* | W165* |  |
| 4.15 | *REG1* |  |  |  |  | *REG1* | S66* |  |
| 4.16 | *HXK2* |  |  |  |  |  |  |  |
| 4.17 |  |  |  |  |  | *CYC8* | W240* |  |
| 4.18 | *HXK2* |  |  |  |  |  |  |  |
| 4.19 | *REG1* |  |  |  |  |  |  |  |
| 4.20 | *REG1* |  |  |  |  | *REG1* | L294* |  |
| 4.21 | *HXK2* |  |  |  |  |  |  |  |
| 4.22 | *REG1* |  |  |  |  | *REG1* | W165* |  |
| 5.1 | *REG1* |  |  |  |  | *REG1* | Q655* |  |
| 5.2 | *GLC7* |  |  |  |  | *GLC7* | Q293P |  |
| 5.3 | *HXK2* |  |  |  |  |  |  |  |
| 5.4 | *REG1* |  |  |  |  | *REG1* | L228I |  |
| 5.5 | *REG1* |  |  |  |  | *REG1* | P797S |  |
| 5.6 | *HXK2* |  |  |  |  |  |  |  |
| 5.7 | *REG1* |  |  |  |  | *REG1* | M443* |  |
| 5.8 | DOM |  |  |  |  | *SNF4* | L78R |  |
| 5.9 | *REG1* |  |  |  |  | *pREG1* | A-166G |  |
| 5.10 | *HXK2* |  |  |  |  |  |  |  |
| 5.11 | *REG1* |  |  |  |  |  |  |  |
| 5.12 | *HXK2* |  |  |  |  |  |  |  |
| 5.13 | *HXK2* |  |  |  |  |  |  |  |
| 5.14 | *HXK2* |  |  |  |  |  |  |  |
| 5.15 | DOM |  |  |  |  |  |  |  |
| 5.16 |  |  |  |  |  |  |  |  |
| 5.17 | DOM |  |  |  |  | *SNF4* | L78R |  |
| 5.18 | *REG1* |  |  |  |  | *GLC7* | E125* |  |
| 5.19 | *HXK2* |  |  |  |  |  |  |  |
| 5.20 |  |  |  |  |  |  |  |  |
| 5.21 |  |  |  |  |  | *TPS2* | R619C | Y |
| 6.1 | *REG1* |  |  |  |  | *REG1* | V349* |  |
| 6.2 | *REG1* |  |  |  |  | *REG1* | E473* |  |
| 6.3 | *REG1* |  |  |  |  | *REG1* | M1* |  |
| 6.4 | *REG1* |  |  |  |  | *REG1* | E473* |  |
| 6.5 | *REG1* |  |  |  |  | *REG1* | Y640* | Y |
| 6.6 | *REG1* |  |  |  |  | *REG1* | K65* |  |
| 6.7 | *REG1* |  |  |  |  | *REG1* | Q293* |  |
| 6.8 |  |  |  |  |  |  |  |  |
| 6.9 | *REG1* |  |  |  |  |  |  |  |
| 6.10 | *HXK2* |  |  |  |  |  |  |  |
| 6.11 |  |  |  |  |  |  |  |  |
| 6.12 | *HXK2* |  |  |  |  |  |  |  |
| 6.13 |  |  |  |  |  |  |  |  |
| 6.14 |  |  |  |  |  |  |  |  |
| 6.15 | *HXK2* |  |  |  |  |  |  |  |
| 6.16 | *REG1* |  |  |  |  |  |  |  |
| 6.17 | *REG1* |  |  |  |  | *REG1* | K175T |  |
| 6.18 | *HXK2* |  |  |  |  |  |  |  |
| 6.19 |  |  |  |  |  |  |  |  |
| 6.20 | *REG1* |  |  |  |  | *REG1* | N624S | Y |
| 7.7 |  |  |  |  |  |  |  |  |
| 7.2 | *REG1* |  |  |  |  | *REG1* | M1R |  |
| 7.3 | *REG1* |  |  |  |  | *REG1* | N21fs |  |
| 7.4 | *HXK2* |  |  |  |  |  |  |  |
| 7.5 |  |  |  |  |  |  |  |  |
| 7.6 | *REG1* |  |  |  |  | *REG1* | G609* |  |
| 7.1 | *REG1* |  |  |  |  | *REG1* | S546* |  |
| 7.8 | *HXK2* |  |  |  |  |  |  |  |
| 7.9 | *HXK2* |  |  |  |  |  |  |  |
| 7.10 | *REG1* |  |  |  |  | *REG1* | L228I |  |
| 7.11 | *REG1* |  |  |  |  | *REG1* | E298* |  |
| 7.12 | *HXK2* |  |  |  |  |  |  |  |
| 7.13 | *REG1* |  |  |  |  | *REG1* | Y640* |  |
| 7.14 | *REG1* |  |  |  |  | *REG1* | P278Q |  |
| 7.15 | *REG1* |  |  |  |  | *REG1* | P278R |  |
| 7.16 |  |  |  |  |  | *FYV10* | S388L | Y |
| 7.17 | *HXK2* |  |  |  |  | *HXK2* | Q251* |  |
| 7.18 | *REG1* |  |  |  |  | *pREG1* | G-113A |  |
| 7.19 |  |  |  |  |  | *FYV10* | S388L | Y |
| 7.20 | *HXK2* |  |  |  |  |  |  |  |
| 7.21 | *HXK2* |  |  |  |  |  |  |  |
| 9.1 | *REG1* |  |  |  |  | *REG1* | Q398* |  |
| 9.2 | *HXK2* |  |  |  |  |  |  |  |
| 9.3 | *HXK2* |  |  |  |  |  |  |  |
| 9.4 | *HXK2* |  |  |  |  |  |  |  |
| 9.5 | *HXK2* |  |  |  |  |  |  |  |
| 9.6 | *HXK2* |  |  |  |  |  |  |  |
| 9.7 | *REG1* |  |  |  |  | *GAL83* | N239K | Y |
| 9.8 |  |  |  |  |  | *GLC7* | Y254C |  |
| 9.9 | *REG1* |  |  |  |  | *REG1* | E195D |  |
| 9.10 | *GLC7* |  |  |  |  |  |  |  |
| 9.11 | *REG1* |  |  |  |  | *REG1* | E195Q |  |
| 9.12 | DOM |  |  |  |  | *SNF1* | Y167D |  |
| 9.13 | *REG1* |  |  |  |  | *REG1* | I279T |  |
| 9.14 |  |  |  |  |  |  |  |  |
| 9.15 | *HXK2* |  |  |  |  |  |  |  |
| 9.16 | *HXK2* |  |  |  |  |  |  |  |
| 9.17 |  |  |  |  |  |  |  |  |
| 9.18 | DOM |  |  |  |  |  |  |  |
| 9.19 | *REG1* |  |  |  |  | *REG1* | L194F |  |
| 9.20 |  |  |  |  |  |  |  |  |
| 9.21 |  |  |  |  |  | *GLC7* | Y254C |  |
| 10.1 | *HXK2* |  |  |  |  |  |  |  |
| 10.2 | *HXK2* |  |  |  |  |  |  |  |
| 10.3 | *HXK2* |  |  |  |  |  |  |  |
| 10.4 | *REG1* |  |  |  |  | *REG1* | Y640* |  |
| 10.5 | *REG1* |  |  |  |  | *REG1* | F468C |  |
| 10.6 | *REG1* |  |  |  |  | *REG1* | Y640* |  |
| 10.7 | *HXK2* |  |  |  |  |  |  |  |
| 10.8 | *REG1* |  |  |  |  |  |  |  |
| 10.9 | *HXK2* |  |  |  |  |  |  |  |
| 10.10 | *REG1* |  |  |  |  | *DCK1* | T592S | Y |
| 10.11 | *HXK2* |  |  |  |  |  |  |  |
| 10.12 | *REG1* |  |  |  |  | *REG1* | N503ins |  |
| 10.13 | *REG1* |  |  |  |  | *REG1* | Y561* |  |
| 10.14 | *REG1* |  |  |  |  | *REG1* | M1I |  |
| 10.15 | *HXK2* |  |  |  |  |  |  |  |
| 10.16 | *HXK2* |  |  |  |  |  |  |  |
| 10.17 | *REG1* |  |  |  |  | *REG1* | S725* |  |
| 10.18 | *HXK2* |  |  |  |  | *HXK2* | G307C |  |
| 10.19 |  |  |  |  |  |  |  |  |
| 10.20 | *REG1* |  |  |  |  | *DCK1* | T592S | Y |
| 10.21 | *HXK2* |  |  |  |  |  |  |  |
| 11.1 | *REG1* |  |  |  |  | *REG1* | Q398* |  |
| 11.2 |  |  |  |  |  |  |  |  |
| 11.3 | *REG1* |  |  |  |  | *REG1* | W227* |  |
| 11.4 | *HXK2* |  |  |  |  |  |  |  |
