# Supplementary material for: A genetic screen reveals a key role for Reg1 in 2-deoxyglucose sensing and yeast AMPK inhibition
Source: PLoS Genet. 2025 Oct 9;21(10):e1011896. doi: 10.1371/journal.pgen.1011896 (PMC12520357; doi:10.1371/journal.pgen.1011896)
Supplement: S3 Table — (DOCX) [file pgen.1011896.s006.docx]

**Supplementary table S3.** Strains used in this study.

| **Strain name** | **Genotype** | **Reference/Origin** | **Figure** |
| --- | --- | --- | --- |
| ySL0066  **WT BY4741** | *Mata; ura3Δ0, his3Δ1, leu2Δ0, met15Δ0* | Euroscarf | 1, 2, 3, 5, 6, 9, S2 |
| ySL0067  **WT BY4742** | *MATα; ura3∆0 , his3∆1, leu2∆0, lys2∆0* | Euroscarf | 1, 3, 4 |
| ySL2856  ***hxk2∆*** | *MATα; ura3∆0 , his3∆1, leu2∆0, lys2∆0 hxk2∆::KanMX* | Euroscarf | 1 |
| ySL1056  ***reg1∆*** | *MATα; ura3∆0 , his3∆1, leu2∆0, lys2∆0 reg1∆::HisMX3* | [1] | 1, 4 |
| ySL2487  ***reg1*-(1-340)** | *MATα; ura3∆0 , his3∆1, leu2∆0, lys2∆0 reg1-Y340** (Spontaneous mutant) | Leon Lab | 1 |
| ySL2857 ***bmh1*∆** | *MATα; ura3∆0 , his3∆1, leu2∆0, lys2∆0 bmh1∆::KanMX* | Euroscarf | 1 |
| ySL2698 ***rod1*∆** | *MATα; ura3∆0 , his3∆1, leu2∆0, lys2∆0 rod1∆::KanMX* | Euroscarf | 1 |
| ySL2488 ***glc7-*Q48P** | *Mata; ura3Δ0, his3Δ1, leu2Δ0, met15Δ0 glc7-Q48P* (Spontaneous mutant) | [1] | 1, 3 |
| ySL3002 ***glc7*-E241K** | *MATα; ura3∆0 , his3∆1, leu2∆0, lys2∆0 art7∆:HisMX3, glc7-E241K* (Spontaneous mutant) | This study | 1 |
| ySL2501 ***hxk2*(1-4)** | *Mata; ura3Δ0, his3Δ1, leu2Δ0, met15Δ0 hxk2-L4** (Spontaneous mutant) | [1] | 1 |
| ySL2482 ***cyc8*(1-320)** | *Mata; ura3Δ0, his3Δ1, leu2Δ0, met15Δ0 cyc8-Q320** (Spontaneous mutant) | [1] | 1 |
| ySL2879 ***reg1*∆** | *Mata; ura3Δ0, his3Δ1, leu2Δ0, met15Δ0 reg1∆::KanMX* | This study | 1, 2, 5, S2 |
| ySL3095 ***reg1∆***  **Dog2-GFP** | *Mata; ura3Δ0, his3Δ1, leu2Δ0, met15Δ0 reg1∆::KanMX, DOG2::GFP-HPH* | This study | 5 |
| ySL3563 **Glc7-GFP Snf1-GFP** | *Mata; ura3Δ0, his3Δ1, leu2Δ0, met15Δ0 SNF1::GFP-KanMX6, GLC7::GFP-URA3* | This study | 6 |
| ySL3564 ***reg1∆***  **Glc7-GFP**  **Snf1-GFP** | *Mata; ura3Δ0, his3Δ1, leu2Δ0, met15Δ0 reg1∆::LEU2, SNF1::GFP-KanMX6, GLC7::GFP-URA3* | This study | 6 |
| ySL3231 ***reg1∆***  **Snf1-GFP** | *Mata; ura3Δ0, his3Δ1, leu2Δ0, met15Δ0 reg1∆::LEU2, SNF1::GFP-KanMX6* | This study | 7, 8 |
| ySL4117  ***SNF1-G53R*** | *Mat a ; his3∆1 leu2∆0 met15∆0 ura3∆0 SNF1(G53R)* (generated by CRISPR) | This study | 2 |
| ySL4317  ***reg1∆*-GFP**  **Hxt3-mCherry** | *Mata; ura3Δ0, his3Δ1, leu2Δ0, met15Δ0 reg1∆::HPH-GFP::HIS3, HXT3::mCherry-KanMX* | This study | 8 |
| ySL4318  **Reg1-GFP**  **Hxt3-mCherry** | *Mata; ura3Δ0, his3Δ1, leu2Δ0, met15Δ0 Reg1-GFP::HIS3, HXT3::mCherry-KanMX* | This study | 8 |
| ySL4345  **Reg1(W165G)-GFP**  **Hxt3-mCherry** | *Mata; ura3Δ0, his3Δ1, leu2Δ0, met15Δ0 Reg1(W165G)-GFP::HIS3, HXT3::mCherry-KanMX* | This study | 8 |
| ySL4346  **Reg1(A54T)-GFP**  **Hxt3-mCherry** | *Mata; ura3Δ0, his3Δ1, leu2Δ0, met15Δ0 Reg1(A54T)-GFP::HIS3, HXT3::mCherry-KanMX* | This study | 8 |
| ySL4347  **Reg1(P231S)-GFP**  **Hxt3-mCherry** | *Mata; ura3Δ0, his3Δ1, leu2Δ0, met15Δ0 Reg1(P231S)-GFP::HIS3, HXT3::mCherry-KanMX* | This study | 8 |

**Reference**

1. Defenouillere Q, Verraes A, Laussel C, Friedrich A, Schacherer J, Leon S. The induction of HAD-like phosphatases by multiple signaling pathways confers resistance to the metabolic inhibitor 2-deoxyglucose. Sci Signal. 2019;12(597):aaw8000. Epub 2019/09/05. doi: 10.1126/scisignal.aaw8000. PubMed PMID: 31481524.
