# Supplementary material for: A genetic screen reveals a key role for Reg1 in 2-deoxyglucose sensing and yeast AMPK inhibition
Source: PLoS Genet. 2025 Oct 9;21(10):e1011896. doi: 10.1371/journal.pgen.1011896 (PMC12520357; doi:10.1371/journal.pgen.1011896)
Supplement: S4 Table — (DOCX) [file pgen.1011896.s007.docx]

**Supplementary table S4.** Plasmids used in this study.

| **Plasmid name** | **Description** | **Reference/Origin** | **Protein expressed** |
| --- | --- | --- | --- |
| pSL21 | pRS416-*pGPD*-mcs-mCherry-*tCYC1*, *CEN*, *URA3* | Leon lab | / |
| pSL388 =  pRS416 | *CEN*, *URA3*. Yeast low copy vector with a *URA3* marker | [1] | / |
| pSL410 | *pDOG2*(1000bp):*lacZ*, 2μ, *URA3* (Yep58-based) | Leon lab  [2] | / |
| pSL559 | *pROD1*(1000bp)-*ROD1*-Flag-*tROD1*, *CEN*, *HIS3* (pRS313-based) | Leon lab | Rod1-FLAG |
| pSL564=  pRS313 | *CEN*, *HIS3*. Yeast low copy vector with a *HIS3* marker | [3] | / |
| pSL575 | p*GLC7*(1000bp)-*GLC7*-GFP-tCYC1, *CEN*, *URA3* (pRS416-based) | Leon lab | Glc7-GFP |
| pSL576 | p*REG1*(1000bp)-*REG1*-Flag-*tROD1*, *CEN*, *HIS3* | This study | Reg1-FLAG |
| pSL608 | yAT1.03 ATP FRET sensor, 2μ, *URA3* | Addgene #13278 | ATP-FRET sensor |
| pSL641 | *pMIG1*(1000bp)-*MIG1*-Flag-*tROD1*, *CEN*, *HIS3* (pRS416-based) | This study | Mig1-FLAG |
| pSL845 | p*REG1*(1000bp)-*REG1*(G160>A)-Flag-*tROD1*, *CEN, HIS3* | This study | Reg1(A54T)-FLAG |
| pSL846 | p*REG1*(1000bp)-*REG1*(A214>G)-Flag-*tROD1*, *CEN, HIS3* | This study | Reg1(R72G)-FLAG |
| pSL847 | p*REG1*(1000bp)-*REG1* (G436>T)-Flag-*tROD1*, *CEN, HIS3* | This study | Reg1(D146Y)-FLAG |
| pSL848 | p*REG1*(1000bp)-*REG1* (G439>A)-Flag-*tROD1*, *CEN, HIS3* | This study | Reg1(D147N)-FLAG |
| pSL849 | p*REG1*(1000bp)-REG1(T493>G)-Flag-*tROD1*, *CEN, HIS3* | This study | Reg1(W165G)-FLAG |
| pSL850 | p*REG1*(1000bp)- *REG1*(T509>C)-Flag-*tROD1*, *CEN, HIS3* | This study | Reg1(I170T)-FLAG |
| pSL851 | p*REG1*(1000bp)-*REG1*(A524>T)-Flag-*tROD1*, *CEN, HIS3* | This study | Reg1(K175T)-FLAG |
| pSL852 | p*REG1*(1000bp)-*REG1*(G583>C)-Flag-*tROD1*, *CEN, HIS3* | This study | Reg1(E195Q)-FLAG |
| pSL853 | p*REG1*(1000bp)-*REG1*(C682>A)-Flag-*tROD1*, *CEN, HIS3* | This study | Reg1(L228I)-FLAG |
| pSL854 | p*REG1*(1000bp)-*REG1*(C691>T)-Flag-*tROD1*, *CEN, HIS3* | This study | Reg1(P231S)-FLAG |
| pSL855 | p*REG1*(1000bp)-*REG1*(T698>A)-Flag-*tROD1*, *CEN, HIS3* | This study | Reg1(V233E)-FLAG |
| pSL856 | p*REG1*(1000bp)-*REG1*(C833>A)-Flag-*tROD1*, *CEN, HIS3* | This study | Reg1(P278Q)-FLAG |
| pSL857 | p*REG1*(1000bp)-*REG1*(T836>C)-Flag-*tROD1*, *CEN, HIS3* | This study | Reg1(I279I)-FLAG |
| pSL858 | p*REG1*(1000bp)-*REG1*(G840>A)-Flag-*tROD1*, *CEN, HIS3* | This study | Reg1(K281E)-FLAG |
| pSL859 | p*REG1*(1000bp)-*REG1*(G840>A A841>G)-Flag-*tROD1*, *CEN, HIS3* | This study | Reg1(H465Y +H467Y)-FLAG |
| pSL860 | p*REG1*(1000bp)-*REG1*(C1393>T T1399>C)-Flag-*tROD1*, *CEN, HIS3* | This study | Reg1(I466M+F468A)-FLAG |
| pSL861 | p*REG1*(1000bp)-*REG1*(A1871>G)-Flag-*tROD1*, *CEN, HIS3* | This study | Reg1(N624S)-FLAG |
| pSL863 | *pSNF1*(500bp)-*SNF1*-mCherry-*tCYC1*, *CEN, URA3* | This study | Snf1-mCherry |
| pSL864 | *pSNF4*(500bp)-*SNF4*-mCherry-*tCYC1*, *CEN, URA3* | This study | Snf4-mcherry |
| pSL865 | *pGAL83*(500bp)-*GAL83*-mCherry-*tCYC1*, *CEN, URA3* | This study | Gal83-mCherry |
| pSL866 | *pSNF1*(500bp)-*SNF1*(T499>G)-mCherry-*tCYC1*, *CEN, URA3* | This study | Snf1(Y167D)-mCherry |
| pSL867 | *pSNF4*(500bp)-*SNF4*(T94>G)-mCherry-*tCYC1*, *CEN, URA3* | This study | Snf4(Y32D)-mCherry |
| pSL868 | *pSNF4*(500bp)-*SNF4*(T233>G)-mCherry-*tCYC1*, *CEN, URA3* | This study | Snf4(L78R)-mCherry |
| pSL869 | *pSNF4*(500bp)-*SNF4*(C382>G)-mCherry-*tCYC1*, *CEN, URA3* | This study | Snf1(P128S)-mCherry |
| pSL870 | *pSNF4*(500bp)-*SNF4*(A487>C)-mCherry-*tCYC1*, *CEN, URA3* | This study | Snf4(S163R)-mCherry |
| pSL871 | *pGAL83*(500bp)-*GAL83*(G535>C)-mCherry-*tCYC1*, *CEN, URA3* | This study | Gal83(G179R)-mCherry |
| pSL872 | *pGAL83*(500bp)-*GAL83*(G673>T)-mCherry-*tCYC1*, *CEN, URA3* | This study | Gal83(D225Y)-mCherry |
| pSL873 | *pGAL83*(500bp)-*GAL83*(C879>G)-mCherry-*tCYC1*, *CEN, URA3* | This study | Gal83(N293K)-mCherry |
|  |  |  |  |

**References**

1. Mumberg D, Muller R, Funk M. Yeast vectors for the controlled expression of heterologous proteins in different genetic backgrounds. Gene. 1995;156(1):119-22. PubMed PMID: 7737504.

2. Defenouillere Q, Verraes A, Laussel C, Friedrich A, Schacherer J, Leon S. The induction of HAD-like phosphatases by multiple signaling pathways confers resistance to the metabolic inhibitor 2-deoxyglucose. Sci Signal. 2019;12(597):aaw8000. Epub 2019/09/05. doi: 10.1126/scisignal.aaw8000. PubMed PMID: 31481524.

3. Sikorski RS, Hieter P. A system of shuttle vectors and yeast host strains designed for efficient manipulation of DNA in *Saccharomyces cerevisiae*. Genetics. 1989;122(1):19-27. PubMed PMID: 2659436; PubMed Central PMCID: PMC1203683.
