# Supplementary material for: A genetic screen reveals a key role for Reg1 in 2-deoxyglucose sensing and yeast AMPK inhibition
Source: PLoS Genet. 2025 Oct 9;21(10):e1011896. doi: 10.1371/journal.pgen.1011896 (PMC12520357; doi:10.1371/journal.pgen.1011896)
Supplement: S5 Table — (DOCX) [file pgen.1011896.s008.docx]

**Supplementary table S5.** Antibodies used in this study.

| **Antibody** | **Description** | **Dilution** | **Reference** |
| --- | --- | --- | --- |
| α-FLAG | Mouse monoclonal against FLAG tag | 1:5000 | #F3156 - Sigma |
| α-GFP | Mouse monoclonal against GFP tag (clones 7.1,13.1) Used in **Figures 3C and 3D** | 1:5000 | #11814460001 - Roche |
| α-GFP | Goat polyclonal against GFP, conjugated to IRDye 800 | 1:2000 | #600-132-215  Rockland Immunochemicals |
| α-Suc2 (Invertase) | Rabbit polyclonal against Invertase | 1:5000 | Colin Stirling |
| α-pAMPK/pSnf1 | Rabbit polyclonal against pT172 of human AMPKα also recognizes pT210 in yeast Snf1 | 1:1000 | #2535  Cell Signaling Technology |
| α-Bmh1 | Rabbit polyclonal against 14-3-3 proteins  (Bmh1 and Bmh2) | 1:15000 | Gift from S. Lemmon, Univ. Miami, USA;  [1] |
| α-RFP | Mouse monoclonal against RFP and RFP derivatives (Used in **Figure 2** to detect mCherry) | 1:2000 | #6G6 Chromotek |
| α-Rabbit IgG | Goat secondary antibody against Rabbit IgG | 1:5000 | #A6154 - Sigma Aldrich |
| α-Mouse IgG | Goat secondary antibody against Mouse IgG | 1:5000 | #A5278 - Sigma Aldrich |

**Reference**

1. Gelperin D, Weigle J, Nelson K, Roseboom P, Irie K, Matsumoto K, et al. 14-3-3 proteins: potential roles in vesicular transport and Ras signaling in *Saccharomyces cerevisiae*. Proc Natl Acad Sci U S A. 1995;92(25):11539-43. Epub 1995/12/05. doi: 10.1073/pnas.92.25.11539. PubMed PMID: 8524799.
