# Supplementary material for: A genetic screen reveals a key role for Reg1 in 2-deoxyglucose sensing and yeast AMPK inhibition
Source: PLoS Genet. 2025 Oct 9;21(10):e1011896. doi: 10.1371/journal.pgen.1011896 (PMC12520357; doi:10.1371/journal.pgen.1011896)
Supplement: Attachment — Submitted filename: PGENETICS-D-25-00740 rebuttal.docx [file pgen.1011896.s009.docx]

PGENETICS-D-25-00740
A genetic screen reveals a key role for Reg1 in 2-deoxyglucose sensing and yeast AMPK inhibition
PLOS Genetics

Dear Dr. Leon,
Thank you for submitting your manuscript to PLOS Genetics. Your manuscript “A genetic screen reveals a key role for Reg1 in 2-deoxyglucose sensing and yeast AMPK inhibition” has been evaluated by three experts. You will see in the enclosed reviews that the reviewers agree that your studies are thorough and of high quality although each of the reviewers raised numerous questions and suggested multiple, primarily editorial, modifications. However, in both the written reviews and in comments to the editors each of the reviewers raised concerns as to the novelty of your conclusions. PLOS Genetics publishes "original research that clearly demonstrates novelty, importance to a particular field, and biological significance". So, the reviewers’ comments raise the concern that your manuscript may be more appropriate for a different journal. On the other hand, we suspect that you and your co-authors may have not convincingly communicated the novel findings of your studies and how they advance the function of Reg1 and/or glucose repression. Thus, we invite you to submit a revised version of the manuscript that addresses the points raised during the review process. If you elect to submit a modified version of Ballin et al. you must satisfactorily address the "novelty" issue in your revised manuscript.

Please submit your revised manuscript within 30 days Aug 28 2025 11:59PM. If you will need more time than this to complete your revisions, please reply to this message or contact the journal office at [plosgenetics@plos.org](mailto:plosgenetics@plos.org). When you're ready to submit your revision, log on to <https://www.editorialmanager.com/pgenetics/> and select the 'Submissions Needing Revision' folder to locate your manuscript file.

Please include the following items when submitting your revised manuscript:

* A rebuttal letter that responds to each point raised by the editor and reviewer(s). You should upload this letter as a separate file labeled 'Response to Reviewers'. This file does not need to include responses to formatting updates and technical items listed in the 'Journal Requirements' section below.
* A marked-up copy of your manuscript that highlights changes made to the original version. You should upload this as a separate file labeled 'Revised Manuscript with Track Changes'.
* An unmarked version of your revised paper without tracked changes. You should upload this as a separate file labeled 'Manuscript'.

If you would like to make changes to your financial disclosure, competing interests statement, or data availability statement, please make these updates within the submission form at the time of resubmission. Guidelines for resubmitting your figure files are available below the reviewer comments at the end of this letter.

We look forward to receiving your revised manuscript.

Kind regards,

Anita K. Hopper
Academic Editor
PLOS Genetics

**Comments to the Authors:**

**Reviewer #1:** Cells can adapt to environmental changes by evolutionarily conserved regulatory pathways. Critically important pathways control the response to fluctuating nutrient levels. One of the best studied pathways of nutrient control is the “glucose repression” pathway that allows yeast cells to switch to growth on poor carbon sources when the major source of nutrients, glucose, becomes limiting. A highly studied kinase, Snf1, homologous to AMPK in humans, controls the de-repression of glucose repressed genes. Surrounding Snf1 are a suite of regulatory proteins whose functions are well known. These include a transcriptional repressor, Mig1, regulatory subunits (Snf4), activating kinases (Elm1 and others), and an essential phosphatase complex (Glc7 and Reg1). Here, a new classical genetic screen (combined with a powerful pinning robot) was performed to identify new regulatory features of this signaling circuit. Mutants resistant to 2DG, a non-metabolizable glucose homolog, were uncovered. Complementation analysis followed by sequencing identified new dominant alleles of key regulatory genes and a large number of recessive alleles. These alleles were confirmed by sequencing and plasmid complementation, and overlaid onto the structures of the protein complexes. A large body of literature was also referred to in order to put the mutations into context. As a result of this effort, a new collection of missense mutations in the Glc7 regulatory subunit Reg1 were identified and characterized. These mutants were resistant to 2DG but did not show relief of other glucose repression phenotypes characteristic of cells lacking Reg1. The mutants were not defective for interaction with Glc7 or 14-3-3 proteins, but were defective in relaying the 2DG signal. The mutants in this class had different phenotypes in various aspects of Hxt transporter internalization and dephosphorylation of the arrestin, Rod1. One mutant W165G when analyzed by quantitative proteomics showed altered protein interactions with Rod1, reduced de-phosphorylation of Snf1, and differences in vacuolar fragmentation.

This is a well thought out, well written, and carefully executed study. A lot of work was done, and much of the model of the Glc7-Reg1-Snf1-Mig1 pathway was verified. The paper was relatively easy to read and understand. It is clear that a new aspect of Reg1 protein was identified that is involved in glucose control. There are a few important questions to address and some minor suggestions.

**We thank the reviewer for their time in evaluating our manuscript and their careful assessment of our work.**

Main points

The major question that comes from reading the paper is what is this new function for Reg1?

**Here, we propose that Reg1 acts as a direct target of a glucose-derived metabolite. Indeed, the mutant W165G is immune to 2DG in terms of signaling but remains sensitive to glucose, suggesting that Reg1 is able to discriminate 2DG from glucose. This is the last sentence of the abstract.**

**We made this appear more clearly in the text, also in response to Reviewer #2’s criticism on the novelty of our work. We stated our hypothesis clearly in l. 444-445. We remodeled the end of the discussion: moving parts, deleting the last paragraph which was a summary that was redundant with the abstract and parts of the discussion, and addition of a paragraph to finish on this hypothesis.**

The authors claim that they have identified a “novel class of missense mutations”; however, their mutations fell into multiple phenotypic classes.

**In summary, the main classes of *REG1* mutants we obtained are nonsense mutation and missense mutations. Whereas the nonsense mutations mimic the *reg1∆* mutant, the missense mutations are globally characterized by a lack of apparent Reg1 function defect in glucose medium (growth, sensitivities to drugs, Snf1 pathway activation etc) although they are 2DG resistant, which we refer to as a “**novel class of missense mutations**”.**

**But indeed, we agree that upon further scrutiny, this class appears later in the study as partially heterogenous, for example when looking at the 2DG response (phosphorylation status of Snf1, Mig1, Rod1, and endocytosis) (Figs 8 & 9). This may reflect different levels of alterations of Reg1 function, or alternatively, separation-of-function alleles as we suggest in the manuscript (mutations that would affect a subset of Reg1 potential substrates), and so far, our data does not allow to discriminate between these possibilities. We propose to change the confusing term “class” which would imply phenotypic homogeneity our text to “a *series*” (Abstract l. 30, l. 137).**

In the abstract, is the conclusion is made that “that 2DG toxicity primarily results from aberrant Snf1 inactivation rather than a direct metabolic disturbance.” What evidence support this conclusion?

**This conclusion stems from the fact that the only defect we found for the Reg1-W165G is a lack of AMPK pathway inhibition in response to 2DG, suggesting that the latter may be causative for the 2DG toxicity. But indeed, we also characterize 2DG-resistant *reg1* mutants in which Snf1 inhibition is observed, so we agree that this conclusion may not apply to all mutants. We thank the reviewer for pointing that out. We have removed that part of the sentence in the abstract as well as the corresponding sentence in the end of the introduction (l. 149).**

Minor points

The introduction was well written. However, a simple model at the beginning might help the reader orient themselves as to the proteins involved.

**Thank you, agreed. We now display a panel in Figure 1 (1A) presenting a working model for the regulation of Snf1 activity by glucose availability and the various actors discussed in the text.**

Figures 5 and 6, there is no mention of statistical tests to show differences. Presumably these tests will be straightforward and will help the reader know which findings are significant and which are not (e.g., Figure 2C).

**Actually, in these figures, the statistical differences are indicated with * or **. No indication means not significant. This is now indicated in the figure legend.**

There are two 14-3-3 proteins, Bmh1 and Bmh2, which one was tested? Is it possible that there is a difference in the interaction with the other Bmh protein?

**In Fig 6C, both Bmh1 and Bmh2 were labeled with the anti-14-3-3 antibodies, hence the two bands in the immunoblot. Because both isoforms run at a similar molecular weight, the quantification (Fig 6D) was made for the overall 14-3-3 signal. Modifications in the panels were made so that this is now clearer.**

“The expression level was not drastically affected by the mutations (Fig 6B), except for the IMFA mutant which consistently showed a twofold, in agreement with previous observations [85].” Add “[twofold] reduction in protein levels”

**Thank you and sorry. This is now corrected.**

Some effort on the figures could make them easier to interpret for the reader. For example:

a. Figure 1C, what does CG refer to?

**Complementation group. This has been corrected in the panel.**

b. Figure 2A, the order is Snf1, Gal83, Snf4, but in Figure 2B, the order is Snf1, Snf4, Gal83. Can the order of panel 2A be moved around to match?

**Sure, this is now corrected.**

c. Can panel 2E be given a title for the protein being modeled?

**Of course - we added a title to the protein model.**

d. Perhaps the letters referring to the panels could be made bold and placed outside of the data in the panels?

**Understood, this has been corrected.**

e. In my version, something is wrong with the error bar in Fig. 7D.

**Sorry – this has now been corrected.**

**Reviewer #2:** This manuscript describes a large-scale screen for spontaneous mutations that confer resistance to the glucose analog, 2-deoxyglucose. The major finding is summarized in the manuscript title where they state that the Reg1 protein plays a key role in 2DG resistance and AMPK inhibition. The strengths of this paper are the thoroughness and scientific rigor of the study. The major weakness of this paper is the lack of novelty. The idea that Reg1 plays a key role in glucose repression and AMPK inhibition has been reported multiple times by many authors going back more than 30 years (Dombek et al 1993; Tu can Carslon 1995). The identification of Reg1 and Hxk2 in screens for 2DG resistance were first reported by Neigeborn and Carlson in 1987 and have been repeated multiple times in a number of labs, including the Leon lab. The big question is what is new here?

**We thank the reviewer for their time in evaluating our manuscript and their careful assessment and critical view of our work. We hope to address the novelty issue raised by the reviewer below.**

Major points
1. The major claim of novelty in this paper is that they have uncovered a new class of reg1 missense mutants.

The prototype, Reg1-W165G fails to impact Snf1 basal activity. For comparison, reg1∆ cells have constitutively active Snf1 kinase while the reg1-W165G allele does not. The known functions of the Reg1 protein are binding to Glc7, Snf1 and Bmh1/2, Glc7 substrate recognition (Snf1, Mig1, Rod1, etc) and maintenance of glucose repression.

The authors do not adequately show the W165G mutant differs from previously identified Reg1 missense alleles in any of these functions. For instance, the basal level of Snf1 phosphorylation is elevated in reg1∆ cells but not in W165G. However, it is not elevated in in the other missense alleles.

**While we agree with the conclusions drawn below by the reviewer from our results, we believe that the major claim is not the identification of a new class of missense alleles, but rather that Reg1 could be central to 2DG sensing, which we think is a novel concept. For example, previous work aimed at identifying such proteins focused on members of the AMPK complex as receivers of the metabolic signal (eg. Milanesi et al. 2021, Ref 97). The origin of the “glucose” signal that inhibits yeast AMPK activity remains elusive despites decades of research, and we believe that the obtention of point mutants in Reg1 that make cells immune to 2DG is a step further in the mechanistic understanding of this question.**

**We made this appear more clearly in the text, also in response to the first comment of Reviewer #1 (see above). We stated our hypothesis clearly in l. 444-445. We remodeled the end of the discussion: moving parts, deleting the last paragraph which was a summary that was redundant with the abstract and parts of the discussion, and addition of a paragraph to finish on this hypothesis.**

**To our knowledge, the only *reg1* missense allele characterized in the past is *reg1-P231L* (Soncini et al. 2020, Ref 46) which is mentioned and discussed in the manuscript (eg. l. 293, 487). This mutant is analogous to one of the mutants we obtained and characterized (*reg1-P231S*) and consequently, much of the conclusions we obtained *in steady-state* *conditions* on this series of mutant were indeed reported for the Reg1-P231L mutant. So strictly speaking, we agree that the concept of a “weak” allele of Reg1 that does not affect Snf1 activity in basal conditions was already published, and maybe the wording of “a new class of mutations” (l. 137) was not appropriate, which we replaced by “a series of mutations” (see also our response to Reviewer #1 on this point).**

**However, we push the characterization further by documenting their ability to respond to 2DG (interaction with Glc7 and Bmh1/2, 2DG6P accumulation and ATP depletion, effect on AMPK pathway inhibition, endocytosis) and expanding these initial observations to many other point mutants. We also show phenotypic variability suggesting different extents of alteration of Reg1 function. In the case of the W165G mutant on which we focused even more, we also see a difference in Reg1 recruitment to the yeast AMPK complex in response to 2DG despite 2DG6P accumulation, which suggests that this mutation alters the 2DG-sensing mechanism. In their paper, Soncini et al. recognize that “*Further characterization of the REG1-P231L allele may allow better understanding of Reg1 protein function in 2DG resistance*” and we believe that our manuscript addresses this point and extends this to other mutants. Finally, the fact that *reg1∆* does not accumulate 2DG6P as much as the WT is also an important finding, as the reviewer recognizes below.**

2. The authors have an assay that directly measures 2DG-6P in cell extracts (Fig 7D and Laussel 2022). One important finding is that reg1 delete mutants do not accumulate 2DG-6P while the missense alleles do. The idea that resistance to 2DG is independent of 2DG-6P accumulation challenges the prevailing model in which 2DG sensitivity is driven by 2DG‑6P accumulation. Why do reg1 delete strains fail to accumulate 2DG-6P?

**Thank you for this comment. An important thing about these 2DG6P content measurements is that they were made shortly (15 min) after exposure to 2DG, as stated in the text (l. 360) and legend (l. 1332). These short-term measurements were made to correlate 2DG6P content with the initial response of the AMPK pathway.**

**However, we did not measure 2DG6P content in cells growing in the presence of 2DG. It could very well be that these mutants (*reg1∆* or Reg1 missense mutants) are able to detoxicate 2DG6P after prolonged incubation, thereby allowing growth. It seems that we did not emphasize this enough in the manuscript, and this has led to confusions when attempting to link growth ability in the presence of 2DG with 2DG6P content (see also Reviewer #3’s comments). We clarified this in manuscript (l. 362, l. 470-471).**

**However, it remains that the *reg1∆* mutant does not accumulate 2DG6P shortly after 2DG treatment, contrary to the other *reg1* mutants we isolated. Based on previously published work, we can hypothesize that 2DG transport is affected (low affinity transporters are either not expressed (Hxt1) or mostly degraded in the vacuole (Hxt3) according to O’Donnell et al. 2015) and detoxification is enhanced through the over-expression of 2DG6P-phosphatases (Defenouillère et al. 2019). The fact that additional hexokinases (Hxk1 and Glk1) that are normally glucose-repressed are also expressed in this mutant may also change 2DG phosphorylation activity. But this remains speculation at this stage. We have completed the sentence initially present in the manuscript (l. 473-475) with this information.**

Why do missense alleles accumulate 2DG-6P but still confer resistance?

**As stated above, the 2DG-6-P content was measured after 15 min of 2DG treatment. We clarified this in l. 362 and l. 471. We do not know to which extent this holds true after longer incubations. This is now mentioned in l. 510-511.**

**However, as stated in the text, we believe that one aspect of 2DG toxicity is the effect that 2DG has on lowering AMPK activity, in addition to its metabolic consequences. As such, we believe that 2DG may cause metabolic alterations in Reg1 missense mutants but in some cases (eg. Reg1-W165G) the inability of 2DG to inhibit AMPK activity could allow cells to dampen the toxic effects of 2DG. This is what we stated in the manuscript (“*in these Reg1 mutants (…) 2DG6P is tolerated and does not trigger a response that is detrimental to cell growth in these conditions*”, l. 504). This hypothesis was previously suggested in Soncini et al. 2020 (“*the common adaptive response to 2DG is to limit the magnitude of the response*”) (cited in l. 507). We completed this paragraph (l. 473-475) so that this hypothesis is now stated more clearly.**

Further the authors do not present the 2DG‑6P accumulation data in the newly characterized hxk2 alleles. Do any of their hxk2 alleles distinguish between glucose and 2DG?

**Indeed, we did not document the *hxk2* alleles. We only sequenced a few to confirm our complementation group-based predictions that these mutants were indeed mutated in *HXK2*, but none of them were re-complemented with WT *HXK2* or re-cloned to prove that the isolated mutations are the only cause of 2DG resistance*.* We did not invest more time into this project. First, there are already many point mutants in *HXK2* described in the literature, many of which already originate from 2DG-resistance screens. Second, one third of the mutants we obtained are in the *HXK2* complementation group, which would have been a lot to investigate, particularly in the context of the already well-described relationship between *HXK2* deficiency and 2DG resistance. Finally, the 2DG6P assay is tedious and expensive. Thus, we did not pursue this further. We only sequenced a few to confirm our prediction that these mutants were indeed mutated in *HXK2.* To answer the reviewer’s question partially, we measured the effect of 2DG on ATP depletion by FRET in a few mutants (figure R1 below). This shows that these behave like an *hxk2∆* strain (see Fig 7E). But whether this is a general rule of the *hxk2* mutants we isolated is uncertain and would require a proper investigation.**

**Figure R1.** Results of ATP FRET measurements performed as described in the manuscript (see **Fig 7E**) (*n=*3*)* revealing a limited ATP depletion after 2DG treatment of 4 isolated mutants (two truncation mutants and two missense mutants).

Note that this was made in the initial strains isolated for the screen which may contain additional mutations than those identified in *HXK2*.

3. The handling of screen “hits” feels inconsistent and difficult to track. A concise flow chart (or summary table) outlining each decision point would greatly improve clarity. Screen progression: show how 195 initial mutants were reduced to 157 by complementation with WT HXK2, REG1, or GLC7. Unassigned mutants (n = 38): list the genes in which these mutations occur and explain why their causative role in 2DG resistance was not confirmed (e.g., whole‑genome sequencing not pursued, technical limitations, etc.).

**Agreed. We now provide a chart (Fig S1) that we hope will solve these interrogations. We thank the reviewer for pointing that out, we believe that this results in an increased clarity of our workflow. This also allowed to describe better the various mutations of class I mutants (see l. 286-289).**

Focused subsets: specify the rationale for choosing the 17 REG1 alleles for detailed analysis.
**We initially studied all missense mutants obtained following complementation group analysis and sequencing. When different substitutions targeted the same residue (eg. P278Q or P278R, E195D and E195Q), we picked only one. This corresponds to our set of 17 mutants. Later sequencing of additional strains revealed new mutant alleles of *REG1* that we had missed in the complementation group analysis (T67N, D661N, P797S) that were therefore not included. We did not study mutant F468C which, from the phenotypes, is a loss-of-function mutation as it carries a mutation in the RHIHF motif for interaction with Glc7. Similarly, point mutations in the start codon (M1R, M1I) which are expected to prevent translation, were not analyzed further. This is now better explained in the text (l.303-309).**

Minor points.
a. Some figures are not accessible to people with color vision issues. For instance, a red-green heat map scale (Fig 5A) and graphs with colors only (no symbols in the lines; Fig 7E).

**Agreed. We changed the colors accordingly.**

b. Fig 4B shows Class I and Class II alleles. Fig 4C shows mutations in Reg1 with missense alleles on top and nonsense alleles on bottom. What are M1* and M1R? This is not explained in the legend.

**Sorry for the lack of clarity. M1* corresponds to a point mutation in the first codon (ATG) causing a stop codon. M1R corresponds to a point mutation in the same codon causing substitution into an arginine residue, presumably preventing translation. This is now added in the legend.**

c. Discussion mentions that the W165G mutant was “still able to dephosphorylate Snf1 in response to glucose” (p.19, line 461). Where is that shown?

**This is shown in Fig 9F (blots) and 9G (quantifications). Unfortunately, Fig 9G was mislabeled (the light green bars should be -Glc + Glc, not -Glc + 2DG – as per the blots in Fig 9F). This is now corrected.**

d. Fig 7D has a misplaced error bar probably belonging to P231S

**Indeed, sorry. This is now corrected.**

e. Fig 9 legend: “Wells were lysed” should be Cells.

**Sorry for the typo. This is now corrected.**

**Reviewer #3:** This is an interesting paper which provides evidence for a crucial role of Reg1 in the initial glucose repression mechanism, based on 2DG toxicity/signaling experiments. The specific REG1 mutant alleles will be very useful for the further elucidation of the initial mechanisms of glucose sensing in the glucose repression pathway.

**We thank the reviewer for their time in evaluating our manuscript and their comments on our work.**

Other comments

Abstract

Please use the term repression only for downregulation at the transcriptional level, repression of a gene. In this sentence:
Among the latter, the Protein Phosphatase 1 (PP1) complex Reg1/Glc7 plays a critical role in repressing Snf1 activity under glucose-rich conditions.’ replace repressing by inhibiting.

**Agreed. This is now corrected.**

The abstract is sometimes confusing because the authors shift between 2DG-induced glucose repression and 2DG-induced toxicity, without addressing the (possible) relationship between the two. There is nothing mentioned in the abstract how 2DG toxicity is related to 2DG-induced glucose repression. Is the latter part of the toxicity mechanism ? If yes, was this already known ?
For instance:
We describe a novel class of REG1 missense mutations, including reg1-W165G, that maintain normal basal Snf1 activity but fail to mediate 2DG-induced Snf1 inhibition, rendering cells effectively immune to 2DG toxicity.

**Thank you for this comment. Previous genetic screens revealed that 2DG is a valuable molecule to identify mutants in the glucose repression pathway. Indeed, *reg1∆* is 2DG resistant whereas *snf1∆* is hypersensitive, i.e. 2DG toxicity resistance with AMPK activity. We and others have shown that high AMPK activity favors 2DG resistance through several mechanisms, notably hexose transporters homeostasis (O’Donnell et al. 2015, Ref 56, Laussel et al. 2022, Ref 50) or Dog2 expression (Defenouillère et al. 2019, Ref 53) and probably other unknown mechanisms.**

**We have modified the abstract to make this appear more clearly.**

Line 99 ‘On the other hand, deletion of HXK2 does not lead to major transcriptional changes [48].’
This is a weird statement since deletion of HXK2 eliminates glucose repression of many genes.

**Yes, this is a common belief but this concept has been recently challenged in Lesko et al. 2023 (Ref 49). According to the authors, the idea that Hxk2 is involved in glucose repression is based on the study of a small number of genes and their RNA-seq data demonstrated that “*HXK2* deletion impacts very few mRNAs” among which *SUC2*, *HXK1* and a few glucose transporters. We have made this discrepancy more evident in the text (l. 112-115).**

Line 104 ‘This response was not observed upon deletion of HXK2, which we proposed to be the main 2DG-phosphorylating enzyme in vivo.’ Would you not expect the opposite effect since glucose phosphorylation by hexokinase should cause the same effect of 2DG phosphorylation ?

**Sorry, it is not clear what the reviewer means here by “opposite effect”. To clarify, in this paragraph, we discuss the fact that 2DG treatment causes Snf1 inhibition, but this does not happen in the *hxk2∆* mutant, suggesting that it is specifically 2DG phosphorylation by Hxk2 that causes Snf1 inhibition, likely through 2DG6P or a derived metabolite as indicated in the text. If the reviewer means that the presence of glucose and its phosphorylation by Hxk1 and Glk1 should mimic 2DG, this is not the case since cells were grown in the presence of glucose and thus Snf1 is already partially inhibited.**

Line 105 ‘Thus, 2DG phosphorylation, i.e. 2DG-6-phosphate itself or a derived metabolite, is central to trigger Snf1dephosphorylation.’ There is also the possibility that active hexokinase itself in some way triggers dephosphorylation of Snf1 as a regulatory protein.

**An interesting result obtained by Randez-Gil et al. (now cited ref 51) shows that overexpression of the 2DG6P phosphatase Dog2 prevents the effect of 2DG on glucose repression. In this condition, Hxk2 would still phosphorylate 2DG but 2DG6P cannot accumulate, favoring the idea that 2DG6P accumulation is central to this regulation. We obtained similar data while looking at the effect of 2DG on endocytosis, which is abrogated by *HXK2* deletion or *DOG2* overexpression (Laussel et al. (Ref 50). We have added a sentence about this for clarifications (l.121-122).**

Line 109 Up to this point the authors have only dealt with the glucose repression mechanism buy which glucose phosphorylation could cause inhibition of SNF1 phosphorylation. Now they will start a screen of 2DG resistant mutants. Is there any evidence that a 2DG resistant mutant would have altered glucose-induced inhibition of Snf1 ? 2DG tolerance can also be caused for instance by higher activity of the DOG1 and DOG2 phosphatases. This aspect should be dealt with.

**Thank you for this important comment. Previous studies cited in the manuscript allowed the isolation of mutants in *REG1 or GLC7*, for instance, causing both 2DG resistance and a loss of glucose-mediated inhibition of Snf1. This is mentioned in l. 92-93. We still added a line about this (l. 125-127) for clarity.**

Line 114 ‘The second one is caused by an increased basal SNF1 activity, either through gain-of-function mutations in all three subunits of AMPK (SNF1, SNF4, GAL83) as previously reported [54] or loss-of-function mutation in the PP1 phosphatase genes, REG1 and GLC7 [45, 50-52].’ Hence, does higher respiration activity overcome 2DG toxicity ? But: Line 118: ‘probably through detoxification mechanisms’: what mechanisms ? Higher activity of DOG1 and DOG2 ?

**Thank you for raising this point. Globally, how an increased Snf1 activity allows 2DG resistance is unclear, and there are currently no links established between respiration and 2DG toxicity. However, our work and that of others (cited in the manuscript) showed that high Snf1 activity leads to increased *DOG1* and *DOG2* expression as well as inhibition of hexose transporters’ endocytosis, which overall may help detoxify the 2DG content of the cells. We have added one sentence to clarify (l. 136-137).**

Line129 ‘consolidate the idea that 2DG toxicity is not due to its direct metabolic effects but rather to an excessive response such as SNF1 inactivation which, when dampened, allows 2DG resistance.’ At first sight this is correct, but if higher Snf1 activity causes higher activity of the DOG1 and DOG2 2DG phosphatases, the idea is no longer correct. In that case it is basically the same as reducing Hxk2 activity, it would just reduce the accumulation of 2DG.

**Agreed, but we believe that this would still be a consequence of high Snf1 activity.**

Line 133 ‘To gain insights into how 2DG is sensed and how it regulates AMPK activity, we performed a 134 genetic screen to isolate 2DG-resistant mutants in glucose-containing medium.’ This is the same issue: the authors link 2DG sensing for initiation of glucose repression to 2DG toxicity. What is the evidence that the two are related ? This should be evidenced more explicitly.

**Thank you. See our comments above concerning l. 109.**

Line 169 ‘We also identified a mutation in TPS2 (clone #5.21), encoding the phosphatase subunit of the trehalose-6-P synthase/phosphatase complex [63] whose deletion was also previously reported to cause 2DG resistance [50]. The identified mutation causes a single substitution (R619C) in the active site of the HAD-like phosphatase domain, which would presumably alter its function, based on structural studies [64].’ Or it could inactivate the enzyme, which causes accumulation of high levels of trehalose-6P, a potent inhibitor of hexokinase.

**Indeed, this is what we meant by “alter its function” – that the activity is somehow lowered or abolished. We changed this into “alter its activity”.**

Line 305 ‘We then studied the phosphorylation of Snf1 and its target Mig1 (Fig 7 A-C). In glucose-grown conditions, the phosphorylation of these proteins was similar in cells expressing WT or mutant alleles of Reg1, in agreement with our hypothesis that Snf1 signaling is not affected (no growth defect, no aberrant DOG2 or SUC2 expression: Fig 4 and 5).’ Does this not indicate that 2DG toxicity is not necessarily related to its glucose repression activity ? Hence, it is important to mention something in the introduction about the presumed connection between the two phenomena.

**Thank you for this comment. In our view, it means that SNF1 does not need to be constitutively active for cells to become resistant to 2DG. We added a comment in the discussion (l. 484-485). However it could be that the lack of 2DG-induced inhibition of Snf1 is sufficient to confer protection against 2DG toxicity. This is now mentioned in the discussion (l. 505-507).**

Line 320 ‘In contrast, the REG1 mutants tested (W165G, A54T, P231S) accumulated 2DG6P to a level that was not significantly different from the WT, suggesting that the lack of 2DG response was not due to an absence of 2DG phosphorylation.’ This is remarkable indeed. There is 2DG phosphorylation but no toxicity. Is it possible that these mutants have higher glucose uptake activity or higher glucokinase activity so that 2DGP is outcompeted by Glu6P ?

**Thank you for this comment. As noted in our response to Reviewer #2, point 2, an important thing about these 2DG6P content measurements is that they were made 15 min after exposure to 2DG, as stated in the text. These short-term measurements were made to correlate 2DG6P content with the initial response of the AMPK pathway.**

**However, we have not measured 2DG6P content in cells growing in the presence of 2DG. It could very well be that these mutants (*reg1∆* or Reg1 missense mutants) are able to detoxicate 2DG6P after prolonged incubation, thereby allowing growth. It seems that we did not emphasize this enough in the manuscript, and this has led to confusions when attempting to link growth ability to 2DG6P content. We have clarified this in manuscript (l. 362-364, l. 470-471).**

**We are sorry about not emphasizing more about this in the initial version of the manuscript.**

Line 325 ‘The phosphorylation of 2DG into 2DG6P is known to be accompanied by a decrease in ATP content, which we observed in reg1∆ cells complemented with WT Reg1 but also with the REG1 mutants tested (Fig 7E).’ Do we have to conclude from this that a drop in ATP or even depletion of ATP does not necessarily compromise growth ?

**As noted above, we have not measured ATP levels upon growth in the presence of 2DG – so we cannot conclude on the ATP content at longer timepoints.**

Line 420 ‘Second, Snf1 is known to inhibit the function of the arrestin-related protein Rod1 in endocytosis [55, 59, 60], and we found that 2DG-induced endocytosis is detrimental to cells in these conditions,’… Endocytosis of what proteins ?

**Work by O’Donnell et al. (Ref 56) and Laussel et al. (Ref 50) revealed that hexose transporter homeostasis is critical for 2DG resistance. This is now precised in the text (l. 467).**
Line 428 ‘First, whereas the reg1∆ strain displays a slow-growth, this was not the case for the missense mutants.’ Slow growth on glucose medium or also on nonfermentable carbon sources ?

**Sorry for the lack of clarity. We meant slow growth on glucose medium, this is now corrected (l. 478).**

Line 450 ‘This also indicates that these Reg1mutants are not resistant because of lack of transport, phosphorylation, or increased detoxification, but rather that 2DG6P is tolerated and does not trigger a response that is detrimental to cell growth in these conditions.’ But there is another important conclusion that the authors overlook: the depletion of ATP caused by the phosphorylation of 2DG and its lack of further metabolism is apparently not a problem for maintenance of cell growth. Or is there no or a reduced drop in ATP in this mutant ? This also raises questions about the intracellular acidification which would normally be caused by ATP depletion because of compromised H+-ATPase activity in the plasma membrane. Would this then not be a problem for cell growth ?

**We hope these questions are addressed by our comments above (comments to l. 320) on the fact that these measurements are made immediately after 2DG treatment, not after long-term incubation. Therefore, we cannot conclude on ATP content of cells growing in 2DG.**

Line 453 ‘This is reminiscent of previous hypotheses that 2DG sensitivity is actually caused by an excessive response’ what type of response is meant ? On Snf1 activity ? Or on something unknown ?

**Sorry again for the lack of clarity. We meant on Snf1 activity indeed. This is corrected (l. 506).**

Line 481 ‘except for its ability to confer growth on 2DG.’ What about its ATP level ? When the cells are able to grow one would except at least some level of ATP in the cells.

**This sentence relates to the Reg1-A54T mutant - its ATP depletion profile is comparable to that of WT cells (Fig 7E), hence our comment that this mutant “was indistinguishable from WT Reg1 on all the aspects that we checked, except for its ability to confer growth on 2DG”.**

Line 488 ‘but normal Snf1 activity in glucose-containing medium.’ Please be specific: ‘but normally inhibited Snf1 activity in …’

**This paragraph has been removed as it was redundant with the abstract and parts of the discussion.**
